# Antibacterial Activity and Mechanism of Action of Black Pepper Essential Oil on Meat-Borne *Escherichia coli*

**DOI:** 10.3389/fmicb.2016.02094

**Published:** 2017-01-04

**Authors:** Jing Zhang, Ke-Ping Ye, Xin Zhang, Dao-Dong Pan, Yang-Ying Sun, Jin-Xuan Cao

**Affiliations:** ^1^Key Laboratory of Animal Protein Food Processing Technology of Zhejiang Province – Department of Food Science, Ningbo UniversityNingbo, China; ^2^Key Laboratory of Animal Products Processing – Ministry of Agriculture, College of Food Science and Technology, Nanjing Agricultural UniversityNanjing, China

**Keywords:** black pepper essential oil, antibacterial activity, *E. coli*, morphological change, intracellular content

## Abstract

The aim of this study was to investigate the antibacterial activity of black pepper essential oil (BPEO) on *Escherichia coli*, further evaluate the potential mechanism of action. Results showed that the minimum inhibition concentration (MIC) of BPEO was 1.0 μL/mL. The diameter of inhibition zone values were with range from 17.12 to 26.13 mm. 2 × MIC treatments had lower membrane potential and shorter kill-time than 1 × MIC, while control had the highest values. *E. coli* treated with BPEO became deformed, pitted, shriveled, adhesive, and broken. 2 × MIC exhibited the greatest electric conductivity at 1, 3, 5, 7, 9, 11, and 13 h, leaked DNA materials at 4, 8, 12, 16, 20, 24, and 28 h, proteins at 4, 6, 8, 10, 12, 14, and 16 h, potassium ion at 0, 0.5, 1, 1.5, and 2 h, phosphate ion at 0.5, 1, 1.5, and 2 h and ATP (*P* < 0.05); 1 × MIC had higher values than control. BPEO led to the leakage, disorder and death by breaking cell membrane. This study suggested that the BPEO has potential as the natural antibacterial agent in meat industry.

## Introduction

Herbs and spices have been applied due to the well-documented sensory properties, special pharmacological functions, and antimicrobial activity (Mata et al., 2007; Park, 2011), where the essential oil (EO) was extracted for meat preserving (Al-Reza et al., 2010; Tajkarimi et al., 2010). The majority of the EOs has been classified as Generally Recognized As Safe (GRAS) by EU standards (Smith and Navilliat, 1997). They are liquid, volatile, and rarely colored, containing a complex mixture of compounds (Bakkali et al., 2008). They are obtained from different plant parts, such as flowers, leaves, seeds, bark, fruits, and roots (Burt, 2004). According to the numbers of isoprene units, EOs are classified as two types: monoterpenes and sesquiterpenes; monoterpenes were the most abundant in EOs components (Nerio et al., 2010). In literature, a large number of biological activities of EOs have been reported, such as antimicrobial, antiviral, antioxidant, antimycotic, antiparasitic, antidiabetic, and anticancer (Reichling et al., 2009).

Meat is an ideal substrate for various spoilage microorganisms, such as *Escherichia coli*, which is recognized as an important cause of food-borne disease and food hygiene indicator bacteria (Bai et al., 2015). Due to the concern of meat shelf-life for both consumers, different kinds of meat preservation techniques have been developed in recent years, among which the natural food preservatives had the potential for application in meat industry. A number of plants can produce natural antibacterial compounds in their tissues defending them against biological hazards (Ryu et al., 2004). Selected plants and their EOs have been evaluated as natural sources for controlling spoilage bacteria during the food storage, so as to extend the shelf-life of food. Recently, the attention of researchers has focused on the antimicrobial activity of EOs, with a strong activity against some bacteria (Bilia et al., 2014). Skandamis and Nychas (2000) reported the antibacterial activity of oregano EO against *Escherichia coli* in eggplant salad. Moghimi et al. (2016) reported the superior antibacterial activity of *Thymus daenensis* EO against *E. coli.*

Black pepper (*Piper nigrum* L.) is a kind of famous spice due to its alluring aroma, typical pungent, and tingling impression (Srinivasan, 2007). Menon et al. (2003) and Zengin and Baysal (2014) have reported that terpenoids have the potential antibacterial activity as the major compounds from black pepper essential oil (BPEO), however, the influence of BPEO on *E. coli* in meat is not still confirmed. Simultaneously, unlike the action of chemical antibiotics, an important characteristic of EO components is their hydrophobicity, which enables them to partition into the lipids of the bacterial cell membrane, disturbing the cell structures, rendering them more permeable, and leading to lysis and leakage of intracellular compounds (Gill and Holley, 2006; Lv et al., 2011; Bajpai et al., 2013). However, to the best of our knowledge, little information is available associated with the antibacterial activity and mechanism of action of BPEO against microorganism.

Therefore, the aim of the present study was to investigate the antibacterial activity and effective concentration of BPEO on *E. coli*, and further evaluate the possible mechanism of action against *E. coli* through kill-time analysis, the changes in bacterial microstructure, the permeability of cell membrane assays, the release of cell constituents (nucleic acids, proteins, potassium, and phosphate ions, ATP), and the membrane potential assays, in order to understand the antibacterial activity and mechanism of action of BPEO better.

## Materials and Methods

### Essential Oils and Chemicals

Black pepper essential oil (pure EO) was purchased from Moellhausen SpA. (Vimercate, Italy). The oil was refrigerated at 4°C in the brown glass bottle. The effect of BPEO on the quality of fresh pork during storage was investigated in our previous study (Zhang et al., 2016). All chemicals used in the study were analytical grade.

### Microorganism and Culture

*Escherichia coli* was chosen to assess the antimicrobial properties. Four strains were isolated from pork, and then biochemical test was carried out by using micro sugar fermentation tube (glucose, lactose, maltose, mannose, and sucrose) and biochemical medium. The strains were identified (data was showed in Supplementary Data Sheet) and maintained on slants of Nutrient Agar (NA) at 4°C in laboratory. The microorganism was cultured in Nutrient Broth (NB) at 37°C for 24 h.

### Antibacterial Assays

#### Agar Disk Diffusion Assay

The antibacterial activity of BPEO was described using the agar diffusion method according to Bajpai et al. (2009) with some modifications. Sterile NA medium was prepared and cooled to 45–50°C before being poured into Petri plates of 90 mm diameter. The disk diameter used was 6 mm (Whatman No. 1) paper. Different dilutions of the EOs were made with 20% of anhydrous ethanol. A loopful of fresh culture of *E. coli* was suspended in a saline solution (0.85% NaCl) and adjusted to a turbidity of 0.5 McFarland standards (about 10^7^ CFU/mL). After solidification, 100 μL inoculum was streaked over the surface of the NA using a sterile cotton swab in order to get a uniform microbial growth on both control and test plates. Under aseptic conditions, the disks were placed on the agar plates and then 5 μL of 0.0, 1.0, 2.0, 4.0, and 8.0 μl/mL BPEO dilutions was put on the disks. A dilution solvent (20% of anhydrous ethanol) was added into the disks on the control plates. Then the plates were incubated at 37°C for 24 h in order to get reliable microbial growth. The diameter of inhibition zone (DIZ, disk diameter included) were measured and recorded in millimeter. The agar disk diffusion tests were performed in triplicate.

#### Determination of Minimum Inhibitory Concentration (MIC)

Minimum inhibitory concentration was determined according to the method described by Weerakkody et al. (2010) with some modifications. Briefly, the suspensions of *E. coli* prepared from overnight broth cultures, were adjusted to the required microbial density (about 10^7^ CFU/mL). BPEO was dissolved in 20% of anhydrous ethanol, then twofold serial dilutions were made in a concentration ranging from 0.125 to 32 μl/mL in 10 mL sterile test tubes containing NB. A 50 μL suspension of *E. coli* was added into the tube. The tube containing only broth and *E. coli* was the negative control. The MIC was determined as the lowest concentration of BPEO that demonstrated no visible growth in tubes after 24 h. All experiments were performed in triplicate.

### Kill-Time Analysis

According to the technique described by Joray et al. (2011), the kill-time curve (survival curve plot) assay method was used to investigate the rapidity of a bactericidal effect or the duration of a bacteriostatic effect of BPEO. Stock solution of BPEO was prepared in 20% anhydrous ethanol. The BPEO at two different concentrations (1 × MIC and 2 × MIC) were added into 100 mL inoculum containing approximately 10^7^ CFU/mL *E. coli*. The inoculum containing only 20% anhydrous ethanol as the negative control was also run. At selected time intervals, the test samples were taken, and plated in Plate Count Agar (PCA) medium (Qingdao Hope Bio-Technology Co., Ltd., China). All plates were then incubated for 24 h at 37°C, and counted the CFU.

### Scanning Electron Microscope (SEM)

To determine the morphological changes of *E. coli* treated with BPEO, SEM studies were carried out as reported by Gao et al. (2011) with some modifications. *E. coli* were incubated in NB at 37°C for 10 h (1 × 10^7^ CFU/mL). Different concentrations of BPEO (control, 1 × MIC and 2 × MIC) were added into the suspensions, respectively. All suspensions were incubated at 37°C for 6 and 12 h, respectively, and then centrifuged at 5,000 × *g* for 5 min at 4°C. The cells were washed three times with 0.1 M PBS (pH 7.4) for 15 min each and fixed in 2.5% (v/v) glutaraldehyde for 2 h at 4°C. The cells were successively dehydrated using 30, 50, 70, 80, 90, and 100% ethanol for 10 min each, and then the ethanol was replaced by tertiary butyl alcohol. After dehydration, the specimens were dried with CO_2_, and sputter-coated with gold in an ion coater for 2 min. Finally, the morphology of the bacterial cell was observed with a SEM (S-3400 N, Hitachi, Ltd., Japan).

### Transmission Electron Microscope (TEM)

The glutaraldehyde-fixed cells described by section “Scanning Electron Microscope” were used for the following treatments. These cells were washed three times with 0.1 M PBS (pH 7.4) for 15 min each and fixed in 2.5% (v/v) glutaraldehyde overnight at 4°C. The cells were washed three times with 0.1 M PBS (pH 7.4) for 15 min each again, and post-fixed with 1% (w/v) osmic acid for 2 h at room temperature, then washed three times with the same PBS. The cells were dehydrated by a sequential graded ethanol (30, 50, 70, and 90%) and then acetone (90 and 100%) for 15 min each. After the dehydration, embedding medium was added into all samples. Stained bacteria were viewed and photographed with a TEM (JEM-1230, Hitachi, Ltd., Japan).

### Permeability of Cell Membrane

The permeability of bacterial membrane is expressed in the relative electric conductivity according to the method described by Diao et al. (2014). *E. coli* was separated by centrifuging at 5,000 × *g* for 10 min, and then washed with 5% glucose until the electric conductivity was near to that of 5% glucose, which indicated the case of isotonic bacteria. The electric conductivity was measured by an electrical conductivity meter (DDS-11D, Shanghai Precision Science Instrument Co. Ltd., China). BPEO at three different concentrations (control, 1 × MIC, and 2 × MIC) were added into 5% glucose; the electric conductivity of the mixtures was measured and marked as L_1_. Then different concentrations of BPEO (control, 1 × MIC, and 2 × MIC) were added into the isotonic bacteria, respectively. After completely mixing, the samples were incubated at 37°C for 10 h; the conductivity was measured per 2 h; it was marked as L_2_. The conductivity of bacteria in 5% glucose treated in boiling water for 5 min was used as the control which was marked as L_0_. The permeability of cell membrane is calculated according to the following formula.

$$\text{Relative}\;\text{electric}\;\text{conductivity}\;(\%)\;=\;\text{100}\;\times \;({\text{L}}_{\text{2}}\;\text{-}\;{\text{L}}_{\text{1}}){\text{/L}}_{\text{0}}$$

### Measurement of Release of 260-nm Absorbing Materials and Proteins

The measurement of the release of 260-nm absorbing materials from *E. coli* according to the method described by Du et al. (2012) with some modifications. Cells from the 100 mL *E. coli* suspension were collected by centrifuging at 5,000 × *g* for 15 min, washed three times with 0.1 M PBS (pH 7.4), and resuspended in PBS (0.1 M, pH 7.4). The 100 mL of cell suspensions was incubated at 37°C under agitation in an environmental incubator shaker (HWS-0288, Ningbo New Jiangnan Instrument Co. Ltd., China) for 4 h in the presence of BPEO of three different concentrations (control, 1 × MIC, and 2 × MIC). Then, the suspensions were centrifuged at 6,000 × *g* for 5 min. The supernatants were diluted with PBS (0.1 M, pH 7.4). Then the absorption at 260 nm of supernatants was measured using a 96-Well Plate Reader M200 (Tecan, Austria) per 4 h. Results were expressed in terms of optical density of 260-nm absorbing materials. In addition, the concentration of proteins in supernatant was determined according to the method described by Xu et al. (2010). All the above steps were repeated and the absorbance at 280 nm was measured. The amount of released protein was calculated by a standard curve using Albumin from bovine serum (BSA).

### Assay of Potassium and Phosphate Ions Eﬄux

The concentration of free potassium and phosphate ions was determined by Lee et al. (2002) with some modifications. The concentrations of free potassium and phosphate ions in bacterial suspensions were measured after the exposure of bacterial cells to BPEO at three different concentrations (control, 1 × MIC, and 2 × MIC) in sterile peptone water (0.1 g/100 mL) for 0, 30, 60, 90, and 120 min, respectively. At each sampled interval, the extracellular potassium and phosphate concentrations were measured by a Kalium/Potassium kit (C001-3, Nanjing Jiancheng Biological Engineering Institute, China) and a phosphorus inorganic kit (C006-3, Nanjing Jiancheng Biological Engineering Institute, China), respectively. Results were expressed as the amount of extracellular free potassium and phosphate (mmol/L) in every interval of incubation.

### Measurement of Extracellular ATP Concentration

To evaluate the influence of BPEO on membrane damage, the extracellular ATP concentrations were measured according to the method described by Lee et al. (2002). The culture of *E. coli* containing approximately 10^7^ CFU/mL was centrifuged for 10 min at 1,000 × *g* and removed the supernatant. The cell pellets were washed three times with 0.1 M PBS (pH 7) and then cells were collected by centrifugation under the same conditions. A cell suspension (10^7^ CFU/mL) was prepared with 9 mL PBS (0.1 M, pH 7), and taken into eppendorf tube for the treatment of BPEO. Then different concentrations (control, 1 × MIC, and 2 × MIC) of BPEO were added into the cell solution. Samples were maintained at room temperature for 30 min, centrifuged at 2,000 × *g* for 5 min, and incubated in ice immediately to prevent ATP loss until measurement. The extracellular (upper layer) ATP concentrations were measured using an ATP assay kit (A095, Nanjing Jiancheng Biological Engineering Institute, China) which comprised ATP assay mix. The ATP concentration of the supernatants, which represented the extracellular concentration, was determined using a 96-Well Plate Reader M200 (Tecan, Austria) to measure the absorption at 636 nm after the addition of 100 μL ATP assay mix to 100 μL supernatant.

### Membrane Potential (MP)

To analyze the effects of BPEO on the metabolic activity of *E. coli*, the MP of the bacteria was measured according to the Rhodamine fluorescence method as described by Baracca et al. (2003) with some modifications. Bacterial cells were incubated in NB medium at 37°C for 24 h. The cell solutions (10^7^ CFU/mL) were added with different concentrations of BPEO (control, 1 × MIC, and 2 × MIC), and incubated in 1 mg/mL Rhodamine123 stock solution with PBS (0.1 M, pH 7) for 3 h. Rhodamine123 was added to a final concentration of 2 μg/mL from the stock solution. The suspensions were washed twice with PBS (0.1 M, pH 7). After placing in dark for 30 min, the samples were completely washed and resuspended in PBS (0.1 M, pH 7). Rhodamine123 fluorescence was measured using a 96-Well Plate Reader M200 (Tecan, Austria) at 530 nm. The data was expressed by mean fluorescence intensity (MFI).

### Statistical Analysis

Statistical analyses were carried out by the One-Way analysis of variance (ANOVA) procedure (Duncan’s Multiple Range Test) of SAS 8.0 software (SAS Institute Inc., Cary, NC, USA) to analyze the difference of DIZ, kill-time, relative electric conductivity, MFI, optical density, the concentration of proteins, potassium, and phosphate ions and ATP among control, 1 × MIC and 2 × MIC groups. The statistically significant level was set as 0.05.

## Results and Discussions

### Antibacterial Activity of BPEO

The antibacterial activity of different concentrations (0.0, 1.0, 2.0, 4.0, 8.0 μL/mL) of BPEO against *E. coli* was qualitatively and quantitatively determined by the presence of inhibition zones. As presented in **Table 1**, the DIZ values for *E. coli* increased significantly (*P* < 0.001) along with the increasing of BPEO concentration. The DIZ values for *E. coli* were shown with range from 17.12 mm to 26.13 mm. The MIC values for BPEO were 1.0 μL/mL for *E. coli*.

**Table 1:** Antibacterial activity of black pepper essential oil (BPEO) against *Escherichia coli* by inhibition zone evaluation.

Concentrations of BPEO (μl/mL)	0.0	1.0	2.0	4.0	8.0
DIZ (mm)	6.12 ± 0.05B^a^	17.12 ± 0.09^b^	19.29 ± 0.09^c^	23.95 ± 0.08^d^	26.13 ± 0.06^e^

*DIZ, diameter values of inhibition zone are presented as mean ± standard deviation, including 6 mm disk diameter. ^abcde^Means with different superscripts, differed significantly for different concentrations of BPEO (*p* < 0.05).*

According to the classification of antimicrobial activity, it is classified into three levels: strong activity (DIZ > 20 mm), moderate activity (12 mm < DIZ < 20 mm), and weak activity (DIZ < 12 mm; Rota et al., 2008; Weerakkody et al., 2010). According to Aligiannis et al. (2001), a classification for the activity is suggested, defining how strong MIC of EOs can hold up to 0.5 μl/mL, moderate for MIC 0.6–1.5 μl/mL, and low for MIC above 1.5 μl/mL. The results in this study showed that BPEO had a strong antibacterial activity against *E. coli*. It could be attributed to its major constituents of sabinene, α-pinene, β-pinene, limonene, β-caryophyllene, and caryophyllene (Menon et al., 2003), which appear to make the cell membrane permeably and disintegrate the outer membrane of Gram-negative bacteria (Burt, 2004).

### Kill-Time Analysis

As was observed in **Table 2**, compared to the control, susceptible *E. coli* treated with BPEO at 1 × MIC level showed a slower decrease in the number of viable cells over the first 12 h period of the test, with the number of viable cells decreased by 16.20% from 6.11 to 5.12 lg CFU/mL (*P* < 0.05), while the number of viable cells decreased obviously from the first hour after cultivation and decreased by 96.73% to 0.20 lg CFU/mL over 24 h of incubation at 2 × MIC (*P* < 0.001). At 6 h, control had the highest number of viable cells; 2 × MIC had lower values than control and 1 × MIC (*P* < 0.05). At 12 and 24 h, 2 × MIC had lowest number of viable cells than control and 1 × MIC (*P* < 0.001 at 12 h, *P* < 0.001 at 24 h, respectively).

**Table 2:** The effect of BPEO on the viability of *E. coli*.

Time (h)	CFU (lg CFU/mL)		
	Control	1 × MIC	2 × MIC
0	6.11 ± 0.38^Ad^	6.11 ± 0.38^Aa^	6.11 ± 0.38^Aa^
1	6.24 ± 0.34^Ad^	5.98 ± 0.34^Aa^	5.91 ± 0.41^Aab^
3	6.41 ± 0.44^Ad^	5.86 ± 0.41^ABa^	5.42 ± 0.43^Bb^
6	7.51 ± 0.47^Ac^	5.59 ± 0.34^Bab^	4.28 ± 0.3^Cc^
12	9.11 ± 0.37^Aa^	5.12 ± 0.38^Bb^	1.23 ± 0.34^Cd^
24	8.24 ± 0.32^Ab^	5.95 ± 0.44^Ba^	0.20 ± 0.18^Ce^

*Values are presented as mean ± standard deviation. ^ABC^Means with different superscripts, differed significantly for different essential oil (EO) treatments (*p* < 0.05). ^abcde^Means with different superscripts, differed significantly for different sampling times (*p* < 0.05).*

The results showed that, a minor amount of BPEO could prolong the lag phase of *E. coli*, while the BPEO at 2 × MIC exerted strong bactericidal activity as evident by the significant reduction in microbial counts and completely inhibition at 24 h exposure, which indicated that BPEO have perfect antibacterial activity against *E. coli*. Similar to our findings, some EOs from plants, which have the major components of terpenoids also exhibited inhibitory effects against various food-borne bacteria (Viuda-Martos et al., 2010; Michalczyk et al., 2012).

### Antibacterial Mechanism Assays

The activity of an EO can affect both the external envelope of the cell and the cytoplasm. The hydrophobicity of the major antibacterial compositions of EOs enables them partition in the lipids of the cell membranes and mitochondria, disturbing their structures, changing their functions and rendering them permeably (Lv et al., 2011). Subsequently, the active components can lead to disrupt the synthesis of some macromolecules, such as DNA, RNA, protein, or polysaccharides, and then cause the death of the cells (Rhayour et al., 2003; Wu et al., 2009). Considering the large number of different groups of chemical compounds present in EOs, it is most likely that their antibacterial activity was not only attributed to one specific mechanism but also several targets in the cell (Skandamis and Nychas, 2001; Carson et al., 2002). Therefore, the surface characteristic parameters, the permeability and integrity of cell membrane were chosen to determine the mode of action of BPEO against *E. coli*.

#### Electron Microscope Observations

**FIGURE 1:**
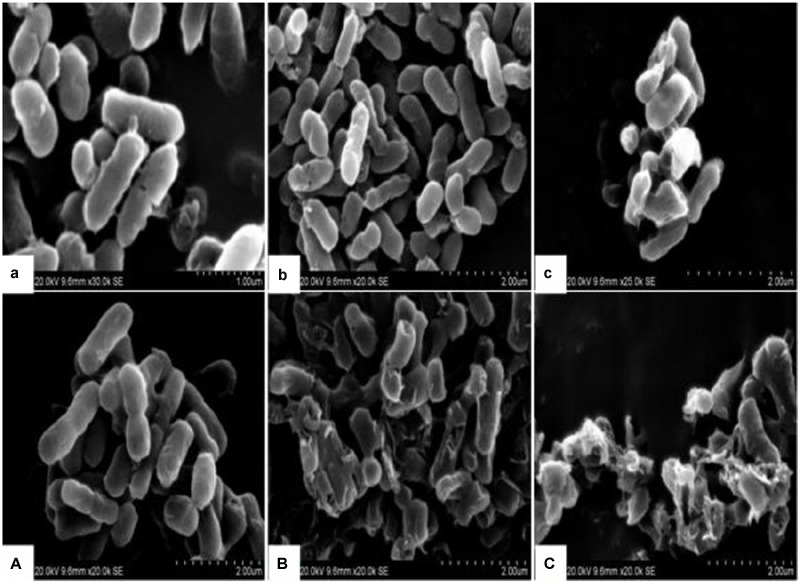
**The scanning electron microscope (SEM) photography of *Escherichia coli* with different black pepper essential oil (BPEO) treatments. (a,A)** Untreated *E. coli* cultured for 16, 22 h, respectively; **(b,B)** *E. coli* treated with BPEO at 1 × MIC for 6, 12 h, respectively; **(c,C)** *E. coli* treated with BPEO at 2 × MIC for 6, 12 h, respectively.

**FIGURE 2:**
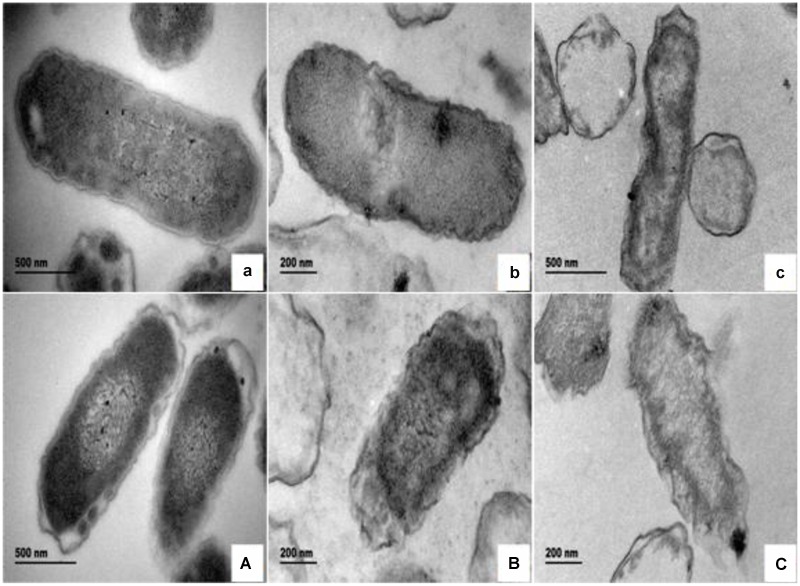
**The transmission electron microscope (TEM) photography of *E. coli* with different BPEO treatments. (a,A)** Untreated *E. coli* cultured for 16, 22 h, respectively; **(b,B)** *E. coli* treated with BPEO at 1 × MIC for 6, 12 h, respectively; **(c,C)** *E. coli* treated with BPEO at 2 × MIC for 6, 12 h, respectively.

The morphological and physical changes of *E. coli* with BPEO treatments at different concentrations were observed by SEM and TEM in **Figures 1** and **2**. SEM images showed that, compared with the untreated controls, the surfaces of the treated *E. coli* underwent obviously morphological changes. Untreated cells were rod shaped, regular, intact, and presented the distinctive characteristics of striated cell wall (**Figures 1a,A**), whereas the cells treated with BPEO became deformed, pitted, shriveled, adhesive to each other; parts of the cell were broken (**Figures 1b,B,c,C**), which might result in the leakage of the contents of the cells. **Figure 2** shows the TEM images of the *E. coli* after treatment with BPEO at different concentrations. It was observed that untreated *E. coli* remained intactly and had a clearly discernible cell membrane with uniformly distributed cytochylema and electron-dense material inside the cell (**Figures 2a,A**). However, the cell wall and cytoplasmic membrane after treatment became uneven and appeared thick; some lysis was seen (**Figures 2b,B,c,C**). Some cells turned from the normal round shape into irregular shapes; parts of the cell wall were broken. It may give rise to the leaching out of nutrient and genetic materials. The changes were more evident with an increase in the concentration and treatment time of BPEO, which was consistent with our results of the SEM and the kill-time study.

The BPEO revealed its inhibitory effect as confirmed by the severe morphological alterations in the cell wall and membrane of *E. coli*. Previously morphological alterations have been observed for various kinds of tested organisms with different EOs (Gao et al., 2011; Paul et al., 2011; Bajpai et al., 2013; Sharma et al., 2013). Moreover, higher BPEO concentration and exposure of longer time could cause impaired membrane structure and swelled cells than control. This observation was in agreement with the kill-time analysis, which showed that BPEO at 2 × MIC could kill much more numbers of *E. coli* at 6 and 12 h than that at 1 × MIC concentration. Cell membrane provide a barrier, which make many cellular processes take place within the cells indispensably, so their damage can cause cell inactivation and/or death. The physical and morphological changes of *E. coli* in this study might be due to the effect of BPEO on the permeability and integrity of the membrane, which would cause the lysis of bacterial cell wall, the expansion and destabilization of the membrane, the separation of cell membrane from cell wall, followed by the loss of intracellular dense materials (Tassou et al., 2000; Bajpai et al., 2009).

#### Permeability of Cell Membrane

**FIGURE 3:**
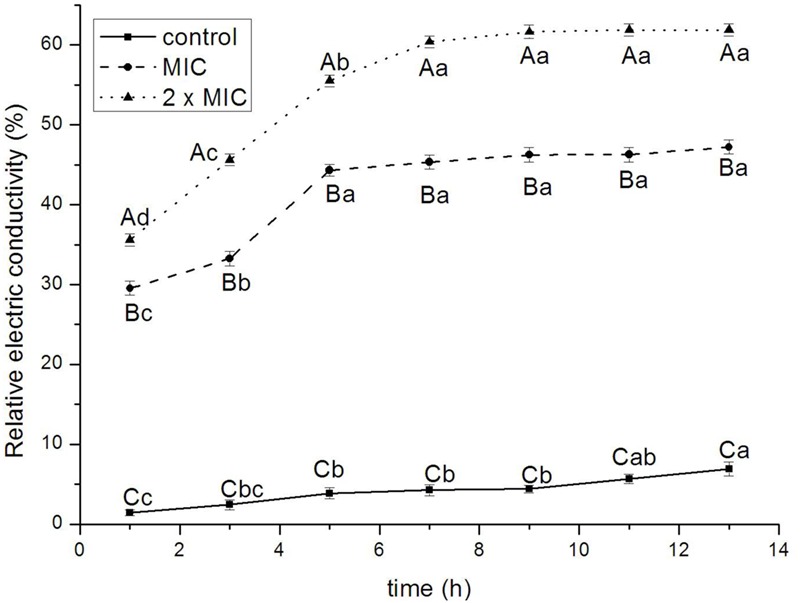
**Effect of BPEO on the impermeability of *E. coli*.** ^ABC^Means with different superscripts, differed significantly for different BPEO treatments (*p* < 0.05). ^abcd^Means with different superscripts, differed significantly for different sampling times (*p* < 0.05).

**Figure 3** showed the effect of BPEO on the permeability of cell membrane of *E. coli*. The relative electric conductivity increased at 9, 11, and 13 h for control (*P* < 0.05), at 1, 3, and 5 h for 1 × MIC (*P* < 0.01) and 2 × MIC (*P* < 0.001) treatments. The relative electric conductivity varied among various treatments; control exhibited the lowest values whereas 2 × MIC remained the greatest value at 1, 3, 5, 7, 9, 11, and 13 h (*P* < 0.01 for 1 and 3 h; *P* < 0.001 for 5, 7, 9, 11, and 13 h).

The antimicrobial mode of action of BPEO was also confirmed on the basis of leakage of the electrolytes from *E. coli* cells when exposed to BPEO at 1 × MIC and 2 × MIC concentrations. The bacterial plasma membrane provides a permeability barrier to the passage of small ions, which are necessary electrolytes to facilitate cell membrane functions, maintain proper enzyme activity and keep the normal metabolism (Diao et al., 2014). It was previously indicated that EOs form channels through membrane by pushing apart the fatty acid chains of the phospholipids, allowing ions to leave the cytoplasm (Burt, 2004). Increase in the leakage of electrolytes indicated the disruption of the permeability barrier. The results in this study showed that the relative electric conductivity of *E. coli* increased rapidly with the increasing treatment time and concentration of BPEO, which meant that the permeability of bacteria membrane would increase correspondingly, then cause the leakage of electrolytes and lead to cell death. Based on these results, we concluded that the increase of cell wall/membrane permeabilization could be related with the hydrophobicity of BPEO.

#### Release of 260-nm Absorbing Materials and Proteins

**FIGURE 4:**
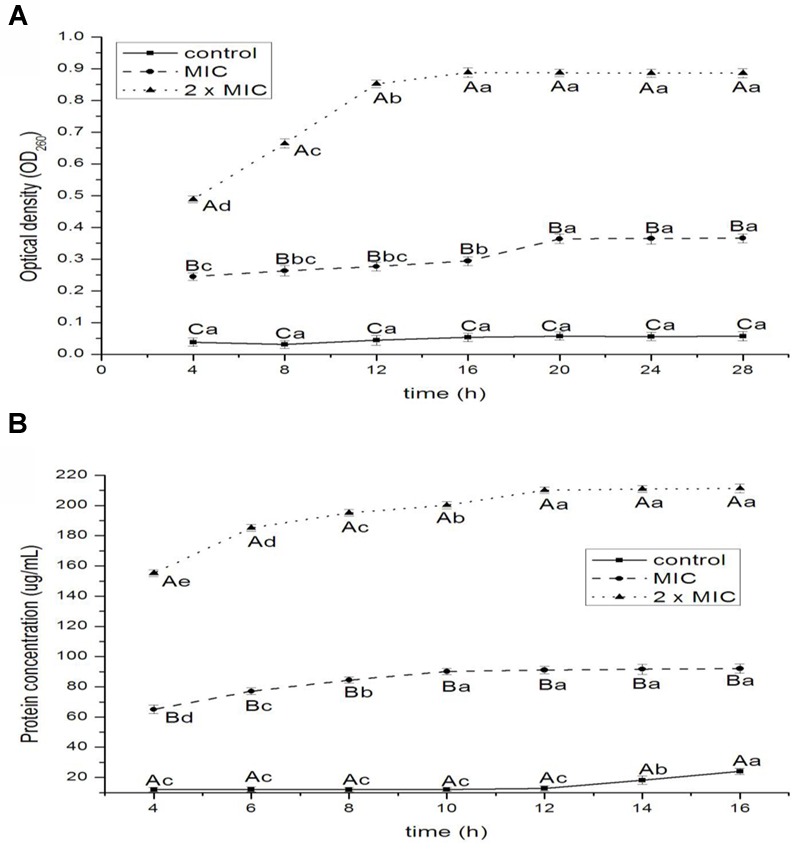
**Release of 260-nm absorbing material (A)** and protein **(B)** from *E. coli* treated with BPEO. ^ABC^Means with different superscripts, differed significantly for different BPEO treatments (*p* < 0.05). ^abcde^Means with different superscripts, differed significantly for different sampling times (*p* < 0.05).

The release of nucleic acids and proteins are shown in **Figures 4A,B**, respectively. The absorbance values for nucleic acids of *E. coli* increased significantly at 16 and 20 h for 1 × MIC (*P* < 0.05), at 8, 12, and 16 h for 2 × MIC (*P* < 0.01) treatments. The OD_260nm_ values of 2 × MIC were higher than that of 1 × MIC; the OD_260nm_ values of control had the lowest value at 4, 8, 12, 16, 20, 24, and 28 h (*P* < 0.05 for 4 and 8 h; *P* < 0.01 for 12, 16, 20, 24, and 28 h). The values of proteins increased significantly at 12, 14, and 16 h for control (*P* < 0.05), at 4, 6, 8, and 10 h for 1 × MIC (*P* < 0.05), at 4, 6, 8, 10, and 12 h for 2 × MIC (*P* < 0.05) treatments. The values of proteins of 2 × MIC were higher than that of 1 × MIC; the values of proteins of control had the lowest value at 4, 6, 8, 10, 12, 14, and 16 h (*P* < 0.05 for 4 and 6 h; *P* < 0.01 for 8, 10, 12, 14, and 16 h).

The 260-nm absorbing materials and proteins, is used as indicative of irreversible damage to the membrane integrity in EOs groups compared to control (Bajpai et al., 2013). The macromolecules of a bacterial cell including nucleic acids and proteins, which reside throughout the interior of the cell and cytoplasm, are the key structural components. Our results showed that the exposure of *E. coli* to BPEO caused the rapid loss of 260-nm absorbing materials and proteins, indicating an irreversible damage to the cytoplasmic membranes, which was supported by the results of the permeability of cell membrane, SEM and TEM. The leakage of nucleic acids and proteins could cause the disorder of function in the synthesis of proteins and DNA materials and the inhibition of bacterial growth.

#### Leakage of Potassium and Phosphate Ions

**FIGURE 5:**
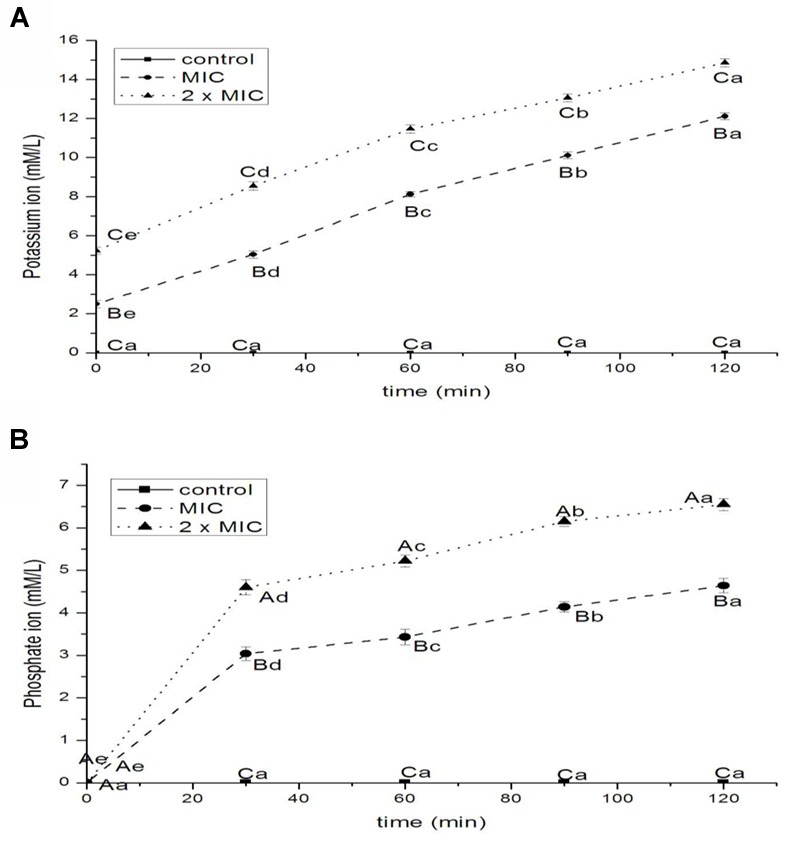
**Leakage of potassium (A)** and phosphate **(B)** ions from *E. coli* treated with BPEO. ^ABC^Means with different superscripts, differed significantly for different BPEO treatments (*p* < 0.05). ^abcde^Means with different superscripts, differed significantly for different sampling times (*p* < 0.05).

The results of release of potassium and phosphate ions from *E. coli* treated with BPEO were shown in **Figures 5A,B**, respectively. The eﬄux of potassium ions from bacterial cells occurred immediately after the addition of BPEO at levels of 1 × MIC (*P* < 0.01) and 2 × MIC (*P* < 0.01) following a steady loss along the specified intervals. The potassium ion values increased rapidly with the increasing concentrations of BPEO (*P* < 0.01 for 0, 30 min; *P* < 0.001 for 60, 90, and 120 min). The eﬄux of phosphate ion from bacterial cells occurred at 30 min after the addition of BPEO at levels of 1 × MIC (*P* < 0.01) and 2 × MIC (*P* < 0.001) following a sturdy loss along the evaluated intervals. The phosphate ion values also increased rapidly with the increasing concentration of BPEO treatment (*P* < 0.01 for 30 and 60 min; *P* < 0.001 for 90 and 120 min).

Potassium levels influence multiple physiological processes, including MP, acid-base homeostasis, fluid and electrolyte balance, glucose metabolism, and blood pressure control (Newman and Cragg, 2012). The internal environment of cells is generally rich in K^+^, so their presence in the extracellular medium is an indication of serious and irreversible cytoplasmic membrane damage (Carson et al., 2002). Phosphates are most commonly found in the form of adenosine phosphates (AMP, ADP, and ATP) as well as in DNA and RNA; they can be released by the hydrolysis of ATP or ADP (Miesel et al., 2003). Therefore, minor changes to the structural integrity of cell membrane can detrimentally affect cell metabolism and lead to cell death (Sharma et al., 2013). Some EOs were recognized to have membrane active properties against several microorganisms, causing leakage of cell constituents, including ions (Turgis et al., 2009). The results of our study showed a significant increase in the leakage of ions. It indicated a disruption of the cell membrane and the disorder of electrolyte balance of *E. coli*, which was consistent with the results of relative electric conductivity.

#### Extracellular ATP Concentration and MP

The effect of BPEO on the extracellular ATP concentration in *E. coli* and MP (expressed by MFI of Rhodamine 123) were presented in **Table 3**. Results showed that the extracellular ATP concentration in control was found to be 1.23 ng/mL. The release of extracellular ATP concentrations of 2 × MIC showed more significant increase than 1 × MIC BPEO (*P* < 0.05). The MFI values decreased rapidly by 37.98% at 1 × MIC level; the MFI of *E. coli* treated with BPEO at 2 × MIC level decreased by 77.95% (*P* < 0.05).

**Table 3:** The effect of BPEO on extracellular ATP concentration and membrane potential (MP) of tested *E. coli*.

Concentration (μl/mL)	ATP (ng/mL)	Mean fluorescence (AU)
Control	1.23 ± 0.15^a^	498.68 ± 7.39^a^
1 × MIC	8.13 ± 0.15^b^	310.01 ± 7.26^b^
2 × MIC	16.10 ± 0.20^c^	111.01 ± 8.89^c^

*Values are presented as mean ± standard deviation in triplicate. ^abc^Means with different superscripts, differed significantly for different means (*p* < 0.05).*

The increase of extracellular ATP concentration exposed to BPEO occurred because of significant impairment in membrane integrity of the tested bacteria by BPEO, which caused the intracellular ATP leakage through defective cell membrane. Burt (2004) has reported that exposure of *Bacillus cereus* cells to carvacrol EO led to the decrease of intracellular ATP. The significant reduction in intracellular ATP could be explained by two mechanisms: the loss of inorganic phosphate across the compromised high permeable cell membrane (Turgis et al., 2009), which is confirmed by the results of the leakage of phosphate ions; or an accelerated hydrolysis due to the attempt of cells to regenerate the electrochemical gradient by PMF driven by the ATPase energy-consuming pump (Nazzaro et al., 2013).

Membrane Potential plays a vital role in the microbial balance and resistance to antimicrobials (Borges et al., 2013). Normally, at physiological conditions, bacterial cells have a negative surface charge due to the presence of anionic groups (e.g., carboxyl and phosphate) in the membrane (Palmer et al., 2007). As an element of the proton motive force (PMF), MP is involved in the generation of ATP (Dimroth et al., 2000). A significant loss of MP renders cells depleted of energy with subsequent death (Ramzan et al., 2010). Any treatments depolarize the cell membrane are deemed to reduce the volume of MP. In this study, the fluorescence intensity was directly correlated with the bacterial MP. Measurements of the MFI of R123 in exponentially growing cells revealed a sharp decrease after the addition of BPEO and indicated a weakening of the PMF. The loss of fluorescence indicates cell membrane depolarization leading to irregular cell metabolic activity and bacteria death.

## Conclusion

Based on the present research, the BPEO possessed a good antibacterial activity against meat-borne *E. coli.* BPEO treatment caused the physical and morphological alterations in the cell wall and membrane of *E. coli*. According to these results, the mechanism of action of BPEO against *E. coli* may be described that, firstly BPEO made a break through the permeability of cell membrane, and then led to the leakage of electrolytes, ATP, proteins, and DNA materials. These changes resulted in disorder, decomposition, and death eventually, which were corresponded to a simultaneous reduction in the number of viable *E. coli*. However, because of the heterogeneous compositions of EOs, it seems unlikely that there is only one mechanism of action or that only one component is responsible for the antimicrobial action. Therefore, further research is still necessary to fully understand the mechanisms, such as the inhibition of food-borne pathogens, the interactions with other food ingredients in order to justify the real applications of BPEO in food practices as a natural antibacterial agent.

## Author Contributions

JZ determined the diameter of inhibition zone, membrane potential and shorter kill-time and writed the manuscript. K-PY determined electric conductivity and did the TEM and SEM. XZ determined the leaked DNA materials and revised the manuscript. D-DP determined the leaked proteins. Y-YS determined the leaked potassium ion. J-XC determined the leaked phosphate ion and ATP.

## Conflict of Interest Statement

The authors declare that the research was conducted in the absence of any commercial or financial relationships that could be construed as a potential conflict of interest.
