# Supplementary material for: Antibacterial Activity and Mechanism of Action of Black Pepper Essential Oil on Meat-Borne Escherichia coli
Source: Front Microbiol. 2017 Jan 4;7:2094. doi: 10.3389/fmicb.2016.02094 (PMC5209337; doi:10.3389/fmicb.2016.02094)
Supplement: Supplementary file 1 [file Data_Sheet_1.DOC]

**Supplements:**

**Strains identifying**

**
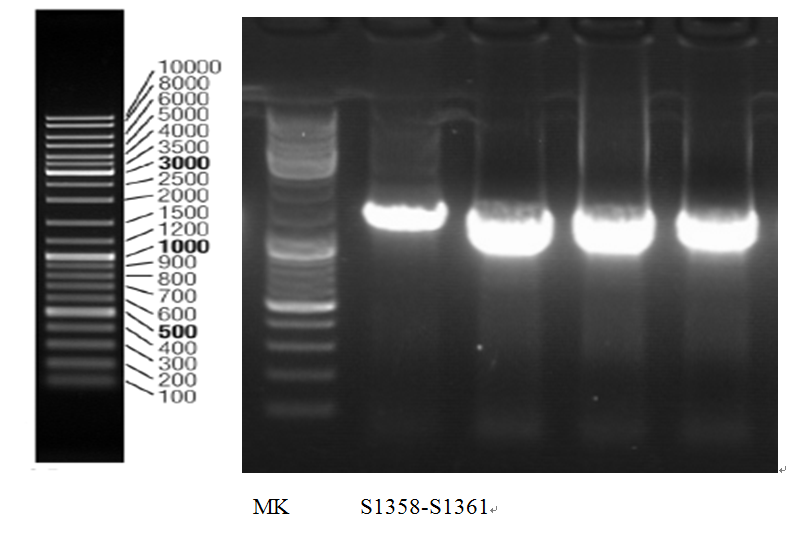
**

**S1359 C 1416bp**

TGCAGTCGAACGGTAACAGGAAGCAGCTTGCTTCTTCGCTGACGAGTGGCGGACGGGTGAGTAATGTCTGGGAAACTGCCTGATGGAGGGGGATAACTACTGGAAACGGTAGCTAATACCGCATAACGTCGCAAGACCAAAGAGGGGGACCTTCGGGCCTCTTGCCATCGGATGTGCCCAGATGGGATTAGCTAGTAGGTGGGGTAACGGCTCACCTAGGCGACGATCCCTAGCTGGTCTGAGAGGATGACCAGCCACACTGGAACTGAGACACGGTCCAGACTCCTACGGGAGGCAGCAGTGGGGAATATTGCACAATGGGCGCAAGCCTGATGCAGCCATGCCGCGTGTATGAAGAAGGCCTTCGGGTTGTAAAGTACTTTCAGCGGGGAGGAAGGGAGTAAAGTTAATACCTTTGCTCATTGACGTTACCCGCAGAAGAAGCACCGGCTAACTCCGTGCCAGCAGCCGCGGTAATACGGAGGGTGCAAGCGTTAATCGGAATTACTGGGCGTAAAGCGCACGCAGGCGGTTTGTTAAGTCAGATGTGAAATCCCCGGGCTCAACCTGGGAACTGCATCTGATACTGGCAAGCTTGAGTCTCGTAGAGGGGGGTAGAATTCCAGGTGTAGCGGTGAAATGCGTAGAGATCTGGAGGAATACCGGTGGCGAAGGCGGCCCCCTGGACGAAGACTGACGCTCAGGTGCGAAAGCGTGGGGAGCAAACAGGATTAGATACCCTGGTAGTCCACGCCGTAAACGATGTCGACTTGGAGGTTGTGCCCTTGAGGCGTGGCTTCCGGAGCTAACGCGTTAAGTCGACCGCCTGGGGAGTACGGCCGCAAGGTTAAAACTCAAATGAATTGACGGGGGCCCGCACAAGCGGTGGAGCATGTGGTTTAATTCGATGCAACGCGAAGAACCTTACCTGGTCTTGACATCCACAGAACTTTCCAGAGATGGATAGGTGCCTTCGGGAACCGTGAGACAGGTGCTGCATGGCTGTCGTCAGCTCGTGTTGTGAAATGTTGGGTTAAGTCCCGCAACGAGCGCAACCCTTATCCTTTGTTGCCAGCGGTCCGGCCGGGAACTCAAAGGAGACTGCCAGTGATAAACTGGAGGAAGGTGGGGATGACGTCAAGTCATCATGGCCCTTACGACCAGGGCTACACACGTGCTACAATGGCGCATACAAAGAGAAGCGACCTCGCGAGAGCAAGCGGACCTCATAAAGTGCGTCGTAGTCCGGATTGGAGTCTGCAACTCGACTCCATGAAGTCGGAATCGCTAGTAATCGTGGATCAGAATGCCACGGTGAATACGTTCCCGGGCCTTGTACACACCGCCCGTCACACCATGGGAGTGGGTTGCAAAAGAAGTAGGTAGCTTAACCTTCGGGAGGGCGCTTACCACTTT

[
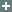
](http://rdp.cme.msu.edu/seqmatch/seqmatch_detailhier.jsp?qvector=204&depth=0&currentRoot=0&querySeq=1&seqid=&num=20)         domain Bacteria  (0/20/1459456) 
[
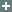
](http://rdp.cme.msu.edu/seqmatch/seqmatch_detailhier.jsp?qvector=204&depth=0&currentRoot=0&querySeq=1&seqid=&num=20)             phylum "Proteobacteria"  (0/20/418265) 
[
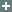
](http://rdp.cme.msu.edu/seqmatch/seqmatch_detailhier.jsp?qvector=204&depth=0&currentRoot=0&querySeq=1&seqid=&num=20)                 class Gammaproteobacteria  (0/20/198197) 
[
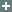
](http://rdp.cme.msu.edu/seqmatch/seqmatch_detailhier.jsp?qvector=204&depth=0&currentRoot=0&querySeq=1&seqid=&num=20)                     order "Enterobacteriales"  (0/20/47931) 
[
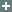
](http://rdp.cme.msu.edu/seqmatch/seqmatch_detailhier.jsp?qvector=204&depth=0&currentRoot=0&querySeq=1&seqid=&num=20)                         family Enterobacteriaceae  (0/20/47931) 
[
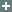
](http://rdp.cme.msu.edu/seqmatch/seqmatch_detailhier.jsp?qvector=204&depth=0&currentRoot=0&querySeq=1&seqid=&num=20)                             genus Escherichia/Shigella  (0/19/11604) 
[S000860484](http://rdp.cme.msu.edu/seqmatch/seqmatch_seqrecorddetail.jsp?seqid=S000860484)     not_calculated 0.990 1329  Escherichia coli; BE42; EF560785
[S002167543](http://rdp.cme.msu.edu/seqmatch/seqmatch_seqrecorddetail.jsp?seqid=S002167543)     not_calculated 0.989 1290  Escherichia sp. II_B13; HM028651

[S002957447](http://rdp.cme.msu.edu/seqmatch/seqmatch_seqrecorddetail.jsp?seqid=S002957447)     not_calculated 0.989 1288  Escherichia coli; M3; JN585664
[S003260464](http://rdp.cme.msu.edu/seqmatch/seqmatch_seqrecorddetail.jsp?seqid=S003260464)     not_calculated 0.990 1214  Escherichia coli; SI-3; AB609042
[S003260466](http://rdp.cme.msu.edu/seqmatch/seqmatch_seqrecorddetail.jsp?seqid=S003260466)     not_calculated 0.992 1191  Escherichia coli; SI-7; AB609044
[S003289542](http://rdp.cme.msu.edu/seqmatch/seqmatch_seqrecorddetail.jsp?seqid=S003289542)     not_calculated 0.991 1293  Escherichia coli; H23; JN129459
[S003289563](http://rdp.cme.msu.edu/seqmatch/seqmatch_seqrecorddetail.jsp?seqid=S003289563)     not_calculated 0.991 1293  Escherichia coli; B13; JN129480
